# Assessment of knowledge regarding tracheostomy care and management of early complications among healthcare professionals

**DOI:** 10.1016/j.bjorl.2021.06.011

**Published:** 2021-08-06

**Authors:** Tooba Khanum, Sadaf Zia, Tahseer Khan, Saima Kamal, Muhammad Nasir Khoso, Javeria Alvi, Arif Ali

**Affiliations:** a Department of Otolaryngology, Head & Neck Surgery (ENT), DIMC (Ohja Campus), DUHS, Karachi, Pakistan; b Department of Pulmonary and Critical Care, DIMC, DUHS (Ojha Campus), Karachi, Pakistan; c Aga Khan University, Department of Critical Care Medicine, Department of Anesthesiology, Pakistan; d Jinnah Post Graduate Medical Centre, Department of Surgery, Karachi, Pakistan; e Dow University of Health Sciences, School of Public Health, Karachi, Pakistan

**Keywords:** Tracheostomy complications, Tracheostomy management, Knowledge assessment

## Abstract

**Introduction:**

Tracheostomy is commonly performed surgical procedure in ENT practice. Postoperative care is the most important aspect for achieving good patient outcomes. Unavailability of standard guidelines on tracheostomy management and inadequate training can make this basic practice complex. The nursing staff and doctors play a very important role in bedside management, both in the ward and in the intensive care unit (ICU) setup. Therefore, it is crucial that all healthcare providers directly involved in providing postoperative care to such patients can do this efficiently.

**Objectives:**

The objective of this study is to assess the knowledge regarding identification and management of tracheostomy-related emergencies and early complications among healthcare professionals so as to improve practice and further standardization.

**Methods:**

Cross-sectional observational study included two hundred and fifty-four doctors and nurses from four large tertiary care hospitals. The questions used were simple and straightforward regarding tracheostomy suctioning, cuff care, cuff management, tube blockage, and feeding management in patients with tracheostomy.

**Results:**

Based on evidence from our study, knowledge level regarding tracheostomy care ranges from 48% to 52% with knowledge scores above 50% being considered satisfactory. Significant gaps in knowledge exist in various aspects of tracheostomy care and management among healthcare professionals.

**Conclusion:**

Our findings demonstrated an adequate knowledge level among health care professionals ranging from 48% to 52% with knowledge scores above 50% being considered satisfactory and revealed that gaps in knowledge still exist in various aspects of tracheostomy care and management.

## Highlights

• Healthcare workers should be well versed in identifying tracheostomy management, its complications and responding accordingly.
• Doctors and nurses (131 = 52%) possessed good knowledge about various aspects of tracheostomy care and management.
• The poorest scores were regarding cuff pressure (38.9%), suction pressure (39.4%) and first response in tube blockade (31.1%).
• Higher scores were found in age group 26 to 30 years (54.2%) and those having 1-3 years of clinical experience (41.2%).
• No statistically significant assoiation of knowledge regarding tracheostomy care was apparent with age, gender or years of practice.

## Introduction

Tracheostomy is a surgical procedure that involves making an incision in the anterior wall of cervical trachea and the overlying skin and soft tissues to relieve restricted airways. It is a procedure which is commonly performed during ENT surgical practice and one of the oldest described surgical procedures in literature.^1^

Its purpose is to provide an airway, in certain cases of obstruction, for prolonged mechanical ventilation in critically ill patients, to provide broncho-pulmonary toilet, airway protection or maintenance and to decrease the dead space of airway.^2^ Tracheostomy is done as either an emergency or elective procedure. Regardless, it is crucial that all healthcare providers directly involved in providing postoperative care to such patients can carry it out efficiently. Moreover, they should also be well aware of any potential risks, complications and their management particularly in immediate life-threatening situations. The complications can be either early or late. The early complications include hemorrhage, tube dislodgement, pneumothorax, and wound infection, while, while late complications include tracheal stenosis, tracheo-esophageal fistula and laryngeal stenosis.^3^

The UK National Confidential Enquiry into Patient Outcomes and Death shows that there is significant morbidity and mortality in tracheostomy patients due to preventable complications. The National Confidential Enquiry into Patient Outcome and Death (NCEPOD) reported that 24% of patients in ICU and 31% of ward patients experienced tracheostomy-related complications.^4^ Annual audit conducted at a tertiary care hospital in Peshawar showed that early complications were 37.5% while late complications were ample 7.5%.^3^

The hospital staff and doctors play a significant role in postoperative care and management of acute and life-threatening complications in such patients, both in the ward and in the intensive care unit (ICU).

Unfortunately, the unavailability of standard guidelines on tracheostomy management and inadequate staff training can make this basic practice much more complex and fearful. Therefore, this study aims to assess the knowledge regarding tracheostomy care among hospital nursing staff and doctors to propose institution-based guidelines to improve practice and further standardization. This study will also help the medicine and nursing domains appropriately design their residency curriculums and ensure adequate delivery of skill at all levels.

## Methods

A cross-sectional descriptive questionnaire-based study was designed to assess knowledge levels in healthcare providers regarding bedside tracheostomy care and management of early complications. Doctors and nurses working in selected government and private tertiary care hospitals in Karachi, including Dow University Hospital (113, 44.8% participants), Jinnah Post Graduate Medical University (25, 9.9% participants), Aga Khan Hospital (73, 29.0% participants), and Liaquat National Medical College (41, 16.3% participants), were included in this study. The calculated sample size is 246 participants using open EPI online software for sample size calculation by taking proportion of knowledge to re-establishing the airway in SpR/Reg group was 20%.^5^ The margin of error was 5%, Confidence interval is 95%.

The questionnaire prepared was designed after a thorough study and review of relevant literature. The questions used were simple and straightforward regarding tracheostomy suctioning, cuff management, tube blockage, and feeding management in patients with tracheostomy.

The inclusion criteria required the subjects to be of either gender or age. Nurses selected were required to have a graduate degree or diploma in nursing sciences and working at either medicine & allied ward, surgery and allied wards, ER, or medical and surgical intensive care units. In contrast, the doctors selected for the study were required to be MBBS degree-holding medical officers, interns, residents, registrars, or senior registrars working in the units, as mentioned above. Ancillary doctors and nurses and those with less than 6 months of clinical experience were excluded from the study.

After obtaining approval for the project from the Institutional Review Board (IRB-1388/DUHS/Approval/2019/), 400 questionnaires were distributed to selected hospitals. An informed and written consent was obtained from each study participant. Two hundred fifty-four filled questionnaires were received. Hence, the study sample comprised of 254 doctors and staff nurses. The required sample was selected using a non-probability convenient sampling technique over six months, from November 2019 to April 2020.

Data was compiled and analyzed in the Statistical Package of Social Sciences (SPSS) version 20.

The descriptive analysis was calculated for quantitative variables, including age and years in practice.

The frequency and percentages were calculated for qualitative variables, that is, qualification, gender, and knowledge. For the mean knowledge score*, descriptive and inferential statistical methods* (independent sample two-tail *t*-test) were used.

## Results

Out of the 254 filled proformas received, 145 (57.5%) were males and 107 females (42.5%). The socio demo-graphic distribution of study population by age, gender, institutes, qualification, and years in practice are displayed in Table 1.

**Table 1:** Socio-demographic profiles of doctors and nurses (n = 254).

Characteristics	n	%
Age		
18–25	78	31.0
26–30	122	48.4
31–35	33	13.1
>36	19	7.5
Gender		
Male	145	57.5
Female	107	42.5
Qualification		
BSN	81	32.1
RN	76	30.2
MBBS	95	37.7
Year in practice		
<1	47	18.7
1–3	103	40.9
4–6	67	26.6
>7	35	13.9

n, Number of people; %, percentage; BSN, Bachelors Of Science in Nursing; RN, Registered Nurse; MBBS, Bachelor of Medicine, Bachelor Of Surgery.

The knowledge scores were categorized as being good if the study participants managed to answer more than 50% of the questions correctly, whereas scores up to 50% or below were considered as being poor. Our data showed that most doctors and nurses (131 = 52%) possessed good knowledge about various aspects of tracheostomy care and management with a mean ± SD of 12.51 ± 2.77.

Our study further revealed that the areas where doctors and nurses had the poorest knowledge regarding bedside tracheostomy care were adequate cuff pressure (38.9%), adequate suction pressure (39.4%), first response in case of tube blockade (31.1%), minimum adequate time for removal of stay sutures (34.8%) and the earliest sign of stomal infections (31.5%). The majority of the health care providers (86.2%) knew that a tracheostomized patient was to be nebulized with a tracheal mask. Table 2 shows doctors’ and nurses’ knowledge regarding the critical steps of bedside tracheostomy care and the percentage of correct responses.

**Table 2:** Knowledge of doctors and nurses about critical steps of bedside tracheostomy care (n = 254).

Subjects were aware about the	F (%)
Ideal time for giving tracheostomy care.	115 (45.3%)
Purpose of interval cuff deflation	156 (61.4%)
Adequate cuff pressure.	98 (38.6%)
Purpose of saline instillation before suctioning.	155 (61%)
Adequate suction pressure.	100 (39.4%)
Adequate suctioning time.	132 (52%)
Method to provide humidification when no special equipment available.	125 (49.2%)
Nebulization with tracheal mask	219 (86.2%)
Checking fenestration of tube.	150 (59.1%)
When to remove fenestrated cannula.	115 (45.3%)
Assessment of tube patency.	141 (55.5%)
First response in case of tube blockade.	79 (31.1%)
Most feared complication in first 48 h.	146 (57.5%)
Minimum adequate time for removal of stay sutures	88 (34.6%)
Prevention of tube dislodgement in mechanically ventilated patients.	132 (52%)
Continuation of per oral feeding in tracheostomized patient unless contraindicated.	149 (58.7%)
First response in suspected trachea-esophageal fistula.	192 (75.6%)
Earliest sign of stomal infection.	80 (31.5%)

F, number of individuals who responded correctly; %, percentage.

Our study also showed that male participants (58%), doctors (42%), and age group ranging from 26 to 30 years (54.2%) had significantly higher knowledge scores. The majority of the participants with good scores have 1–3 years of clinical experience (41.2%), as displayed in Table 3. However, we did not find any statistically significant association of knowledge regarding tracheostomy care with age, gender, qualification, and years of doctors’ and nurses’ practice.

**Table 3:** Association of knowledge scores with socio-demographic characteristics of doctors and nurses (n = 254).

Knowledge of tracheostomy	Poor		Good		p-Value
Statistics	n	%	n	%	
Age					0.178
18–25	44	36.4%	34	26.0%	
26–30	51	42.1%	71	54.2%	
31–35	15	12.4%	18	13.7%	
>36	11	9.1%	8	6.1%	
Gender					0.874
Male	69	57.0%	76	58.0%	
Female	52	43.0%	55	42.0%	
Qualification					0.333
BSN	41	33.9%	40	30.5%	
RN	40	33.1%	36	27.5%	
MBBS	40	33.1%	55	42.0%	
Year in practice					0.897
<1	23	19.0%	24	18.3%	
1–3	49	40.5%	54	41.2%	
4–6	34	28.1%	33	25.2%	
>7	15	12.4%	20	15.3%	

n, Number of people; %, percentage; BSN, Bachelors Of Science in Nursing; RN, Registered Nurse; MBBS, Bachelor of Medicine, Bachelor Of Surgery.

Our findings also revealed that the majority of our study participants agreed that professionally developed guidelines and Educational programs/CME/Workshops would serve to improve knowledge and practice regarding tracheostomy care.

## Discussion

Tracheostomy is regarded as an important lifesaving procedure in many conditions and has become a well-established procedure with more specific indications.^6^ Over the last 100 years, with further refinements in technique and equipment, the rates of morbidity and mortality associated with tracheotomy have continued to decline^7^ and the indications for the procedure have evolved with our ability to keep critically ill patients alive, such that almost two thirds of tracheotomies are now performed on intubated intensive care unit (ICU) patients.^8^ There are two approaches to tracheostomy: open surgical tracheotomy (ST) and percutaneous dilatational tracheostomy (PT). ST has traditionally been undertaken by Ear, Nose, and Throat (ENT) (Otorhinolaryngology) surgeons. With the increasing use of PT, a wider range of healthcare providers are now directly involved in the care of patients with a tracheostomy and need to be familiar not only with tracheostomy care, but also with the techniques of decannulation and management of acute and life-threatening complications.^5^ Therefore, in this study, we have attempted to assess the knowledge of tracheostomy care and management of specific tracheostomy related emergencies and early complications among hospital nursing staff and doctors to identify gaps in knowledge. This study will also serve as a useful reference article when designing medical and nursing residency curriculums. It will also be useful in proposing institution-based guidelines regarding tracheostomy care and management.

Tracheal suctioning is an essential aspect of effective airway management. However, this has many associated risks and complications, ranging from trauma and hypoxemia to, in extreme cases, cardiac arrest and death.^9^ In a study conducted by Varshney et al.^10^ 315 nurses were evaluated about their tracheal suctioning practices, and subsequently completed a knowledge based questionnaire. The findings demonstrated a poor level of knowledge among the majority of subjects. Similar findings were found in a study conducted by Day et al.,^9^ as many nurses failed to demonstrate an acceptable level of competence for knowledge and practice. We saw similar findings in our study, where only 39.4% of our study participants knew about adequate suction pressures, and only 52% correctly answered about the adequate tracheal suction time.

A difference of opinion is found among health care professionals and in the literature concerning instillation of normal saline before suctioning. Liquefying secretions are thought necessary in such patients to ensure easy and rapid removal through suctioning. Instilling a few cc of sterile 0.9% saline is a common clinical practice, though a meta-analysis conducted by Wang et al. revealed that NS instillation does not provide clinical benefits in heart rate, blood pressure, and pH in patients undergoing endotracheal intubation or tracheostomy, and it can even lead to decreased oxygen saturation 5 min after suctioning. According to this review, evidence so far suggests that NS instillation should not be used in clinical practice.^11^ While a survey of suctioning techniques and Airway Management Practices conducted by Sole et al.^12^ which included 1665 nurses and respiratory therapists from 27 different areas in the United States, reported that 74% centers had a protocol which recommend instillation of isotonic sodium chloride solution for thick secretions while suctioning, 61% of our study participants also agreed to its usefulness.

Negative pressure is applied to draw out tracheal secretions. The amount of negative pressure applied while suctioning is of great importance as less pressure might not be effective and excessive pressures can cause mucosal damage, trauma and pulmonary atelectasis.^13^ Most of the literature recommends that the pressure range during tracheal suctioning should be from 70 to 150 mmHg.^9^, ^14^ Our study showed that only 38.6% of health care professionals were aware of the adequate range cuff pressures, hence highlighting the lack of basic concepts of tracheostomy care.

It is important that all healthcare workers directly involved in the postoperative care of tracheostomy patients can provide proper tracheostomy care, are aware of the potential tracheostomy-related complications, and can manage these complications, particularly in an immediate life-threatening situation.^5^ According to a study conducted by Onakoya et al.^15^ the complications of tracheostomy fall into three groups: (1) infection: pneumonia or other infection; (2) tube blockage or displacement; (3) assorted. In cases of tracheostomy tube bloackage, continued attempts at ‘rescue’ ventilation will not be effective as the airway is obstructed and the tracheostomy tube should be removed.^16^ Following tracheostomy tube removal, reassessment at both airways (mouth and trachea) is required, ensuring oxygen is reapplied to face and stoma.^17^, ^18^, ^19^ These actions may resolve the airway problem and if the patient is breathing and improving, ABCDE assessment continues. Definitive management of the airway (re-insertion of a tracheostomy or oral tube) is not necessarily required immediately if the patient is not hypoxic.^16^ When asked about their immediate response in the case of a blocked tracheostomy tube, most of our study participants were surprisingly in favor of removing the obstruction. As per our data, this was the weakest area of understanding in tracheostomy care and management, with only 31.3% correct answers.

The use of stay sutures in tracheostomy is proposed. They can be of help during the performance of the operation and can be of even greater benefit after it. If the tube is displaced from the trachea in the early postoperative period traction on these sutures permits rapid reintubation.^20^ Lateral tracheal stay sutures at the third or fourth tracheal rings can provide lateral traction and stabilization and help to define the stoma.^21^ Stomal maturation usually is complete after 5 days and the first tracheostomy tube change may be safely performed by the surgical team. The original tube is removed; stoma wiped clean with sterile gauze and the new tracheostomy tube is placed. Soft tracheostomy ties are placed snugly around the neck and stay sutures removed.^22^ In a study conducted by Casserly et al.^5^ among the participants interviewed, 100% of the ENT subgroup was familiar with the concept of stay sutures compared with 23% of the anesthesia group, 37% of the ICU nurses group, and 31% of the ENT ward nurses group. When we asked health care professionals what the minimum adequate time for removal of stay sutures was, only 34.6% of our study participants managed to answer correctly delineating another area of tracheostomy care where a gap in knowledge exists.

Most of the complications associated with tracheostomy are largely preventable and could be reduced or avoided by strict adherence to the careful performance of the procedure as well as to postoperative care.^23^, ^24^ According to Onakoya et al.^15^ the most common complications of tracheostomy was infection representing 43% of all complications^15^ which is in conformity with the literature.^23^ Stomal infection can be a very troublesome complication in its own right^25^, ^26^ apart from whatever role it might have in parenchymal lung infection’s pathology. Furthermore, local infection may influence the later development of tracheal stenosis.^27^ According to Friedman et al.^28^ a wound/stoma infection was present when there was purulent drainage from the site. Crofts et al.^29^ defined stomal infection as inflammation and purulent drainage requiring antibiotic therapy. According to Holdgaard et al.^30^ stomal infection was quantified as the distance in millimeters that cellulitis extended from the stoma in conjunction with purulent sputum. When asked, “what was the earliest sign of stomal infection in a patient with a tracheostomy?”, only 31.8% of the study participants managed to answer correctly, identifying yet another weak area of understanding in tracheostomy care and management.

## Conclusion

Our findings demonstrated an adequate knowledge level among health care professionals ranging from 48% to 52% with knowledge scores above 50% being considered satisfactory and revealed that gaps in knowledge still exist in various aspects of tracheostomy care and management.

## Limitations

Our study had a few limitations that need to be mentioned. Firstly, our study is based on convenience sampling, and only those doctors and nurses who were willing to participate were included. Thus, our findings might be subjected to a degree of volunteer bias. Second, owing to the gender distribution at the hospitals, we had a higher percentage of males included. Third, our study was confined to the theoretical assessment of doctors and nurses. Had we included a simulation-based exam and the theoretical assessment, the evaluation of both knowledge and practice would have given a much clearer picture of tracheostomy care and management of its early complications amongst health care professionals.

### Implications for clinical practice

Based on the findings of our study, the following implications can be made:

A similar study can be replicated on a larger sample size with a simulation-based exam and theoretical assessment to obtain an even more precise evaluation of knowledge and practice levels regarding tracheostomy care and management.

Forming specific guidelines and management algorithms that are readily available at the bedside of every tracheostomy patient.

A study incorporating a teaching intervention is therefore recommended to refine the knowledge, competence, and understanding of nurses and doctors involved in the care of patients with tracheostomy.

Our study outlines the need for emphasizes the importance of continuing medical education classes and simulation-based exercises for concerned health care providers in order to achieve lesser gaps in knowledge base health care providers.

## Conflicts of interest

The authors declare no conflicts of interest.
